# Dynamic Changes in Microbial Communities in Oil Reservoirs Under a Long-Term Bio-Chemical Flooding Operation

**DOI:** 10.3390/microorganisms13102246

**Published:** 2025-09-25

**Authors:** Gui-Na Qi, Guo-Jun Li, Yi-Fan Liu, Lei Zhou, Ya-Qing Ge, Jin-Feng Liu, Shi-Zhong Yang, Ji-Dong Gu, Bo-Zhong Mu

**Affiliations:** 1 State Key Laboratory of Bioreactor Engineering, School of Chemistry and Molecular Engineering, East China University of Science and Technology, Shanghai 200237, China; qiguina@mail.ecust.edu.cn (G.-N.Q.); liguojun@mail.ecust.edu.cn (G.-J.L.); liuyifan@ecust.edu.cn (Y.-F.L.); leizhou@ecust.edu.cn (L.Z.); meor@ecust.edu.cn (S.-Z.Y.); 2 Daqing Huali Biotechnology Co., Ltd., Daqing 163000, China; shapigazi123@126.com (Y.-Q.G.); ljf6713@sina.com (J.-F.L.); 3 MOE Engineering Research Center of Microbial Enhanced Oil Recovery, East China University of Science and Technology, Shanghai 200237, China; 4 Environmental Science and Engineering Program, Guangdong Technion—Israel Institute of Technology, 241 Daxue Road, Shantou 515063, China; jdgu.aeb@gmail.com

**Keywords:** petroleum reservoir, bio-chemical flooding, microbial community, dynamic change

## Abstract

Huge amounts of water and chemicals have been injected into subsurface oil reservoirs in secondary and tertiary oil recovery processes. Although the effects of injected water and chemicals on microbial communities have been investigated, knowledge about their long-term dynamic changes in oil reservoirs remains limited. To address this gap, we used 16S rRNA sequencing from cDNA and chemical analysis to track the dynamic changes in microbial communities in oil reservoirs under a long-term flooding operation over three years and five months using bio-chemical flooding in the Daqing Oilfield, China. Researchers observed dynamic changes in microbial composition and diversity during the flooding process. Long-term bio-chemical drainage leads to alterations in dominant bacterial community structure, with a decrease in methanogenic archaeal abundance. Bacterial metabolic functions remained stable, but archaeal functions changed notably. Our results indicate that the microbial community and its functions in the oil reservoirs have experienced significant dynamic changes under the long-term flooding intervention of bio-chemical flooding, which opens up a new window for further understanding the impact of injected water and chemicals on microbial community in oil reservoirs and expands our knowledge about the role of microbial community changes in reservoirs under the flooding process.

## 1. Introduction

Petroleum reservoirs are unique ecological environments in the deep subsurface biosphere, with multiphase fluids containing oil, gas, and water, in addition to a high temperature, high pressure, and high salinity, presenting as complex oligotrophic ecosystems [1]. It is widely believed that crude oil is not a preferred habitat for microbes because of its potential toxicity and high hydrophobicity [2]. Since the first report on a sulfate-reducing bacterium isolated from production water [3], oil reservoirs, petroleum-related microbes, and their metabolic activities have received much attention due to their important role in biogeochemical cycles in subsurface ecosystems. With the development of biotechnology and molecular biology techniques, a large amount of hydrocarbon-degrading, surfactant-producing, and anaerobic methane-producing strains have been isolated or detected in different oil reservoirs worldwide [4,5].

Unlike the bulk of the deep biosphere, due to the complexity of oil reservoir system and the diverse injection for production technology, the in situ ecosystem of the oil reservoirs was altered significantly physiologically and phylogenetically, and the composition of the microbial community in oil reservoirs was altered remarkably by different injection methods [6]. Specifically, different injection methods, such as microbial-enhanced oil recovery (MEOR), indigenous microbial recovery technology, and ASP-flooding and polymer-flooding oil recovery technologies, have been shown to reshape reservoir microbial community composition [7,8,9]. A study of microorganisms in oil reservoirs under different replacement methods showed that an increase in oil production was associated with an increase in functional microorganisms, especially during MEOR technology operation [10,11]. However, existing research predominantly focuses on short-term microbial responses [12,13,14]. A critical knowledge gap remains, and there is a lack of systematic studies on microbial community dynamics under long-term (≥3 years) bio-chemical composite flooding.

Notably, previous studies on reservoir microorganisms have only explored total microbial communities via 16S rRNA gene sequencing, failing to capture the dynamics of metabolically active microbes that directly mediate functional processes. This limitation hinders the accurate linking of microbial community shifts to understand the functional response to the flooding.

To address this gap, we investigated the dynamics of metabolically active microbial communities (analyzed via cDNA-based 16S rRNA sequencing, targeting active cells) in reservoirs of the Daqing Oilfield over a 3-year-and-5-month bio-chemical flooding period. The specific objectives are to characterize the temporal succession of active bacterial and archaeal communities during different flooding phases, identify key physicochemical factors driving the enrichment of active functional taxa, and predict the shift in microbial metabolic functions (KEGG pathway analysis: metabolic functions were predicted using PICRUSt2 ) and link it to flooding-induced environmental changes.

The present study is pioneering in its exploration of active microbial community dynamics under long-term bio-chemical composite flooding, a time scale which surpasses that of existing short-term studies. Furthermore, it utilizes cDNA-based sequencing to target active microbes, thereby addressing the limitation of traditional 16S rRNA gene sequencing that confounds active and inactive cells. Consequently, this provides a theoretical foundation for the optimization of long-term bio-chemical flooding strategies.

## 2. Materials and Methods

### 2.1. Site Description and Sampling

This study was conducted at the sandstone reservoir in the Daqing Oilfield in the northeast of China, which has been subjected to water flooding since 2019. The block chosen for monitoring includes 154 production wells and 141 injection wells. Each production well (A, B, C) is surrounded by 4 injection wells, with well spacing being 125 m. The average thickness of the oil layer is 8.60 m, with an average temperature of 49.4 °C, a formation pressure of 10.5 MPa, and average permeability of 0.219 μm^2^. The density of the crude oil is 0.87 g/cm^−3^, with an oil viscosity of 28.4 mPa·s on the ground. A new mixture consisting of surfactant, lipopeptide biosurfactant, Na_2_CO_3_, and hydrolyzed polyacrylamide was formulated. The dosage of the components was optimized to be as follows: petroleum sulfonate (0.2%, *w*/*v*), lipopeptide biosurfactant (0.2%, *w*/*v*), and Na_2_CO_3_ (1.2%, *w*/*v*). The concentration of hydrolyzed polyacrylamide (HPAM, molecular weight 1.2–1.6 × 10^7^ Da) was determined to be 2000 mg/L to achieve better mobility for the oil–water ratio. When flooding with this mixture was initiated from August 2020, the water cut of a single well was in the range of 83.26–98.75%. Microbial communities and physicochemical factors of the sampled oil reservoir were constantly monitored over the period of the report, from July 2020 to December 2023. Production fluid samples were collected from the production wells affected by three injection wells in the petroleum reservoirs under a combination of bio-chemical flooding, and the temperature of the strata was 45–47 °C, the pH was 7.73–9.40, and the samples were oil–water mixtures. To avoid disturbance from other nearby injection wells, we selected sampling sites from production wells influenced solely by the three studied injection wells, yielding one production well per injection well, namely, N7-10 (Well A), N7-21(Well B), and N7-D3 (Well C).

The injection systems include surfactant, Na_2_CO_3_, and hydrolyzed polyacrylamide. Among them, the surfactant is composed of petroleum sulfonate and lipopeptide microbial cultures in equal proportions (1:1). The microbial culture was a pure culture of Bacillus subtilis producing lipopeptide biosurfactant, with a biosurfactant concentration of 1.5–2.0 g/L and a surface tension of 27.6 ± 1.0 mN/m, as measured using a fully automatic tensiometer JK99C (Shanghai Zhong Chen Electric Co., Ltd., Shanghai, China) by the du Nouy ring method [15,16]. Samples were collected from the production wellheads into 25-L sterilized plastic bottles (adding 20% RNA stabilizing agent composed of ethanol and Trizol 95:5 *v*/*v*; Thermo Fisher Scientific, Waltham, MA, USA) to capacity; the bottles were then securely sealed with screw caps. The bottles were transported back to the laboratory for further processing and analyses. A total of 50 L of production water was collected for RNA extraction. Microbial cells were collected from each water sample by centrifugation at 4 °C for 20 min at 10,000× *g* in a high-speed centrifuge. The collected bacterial cells were stored in a refrigerator at −80 °C before PCR amplification [17]. A total of 7 sample groups were collected (S_0_ toS_6_), with each group consisting of 3 individual samples (one from each production well—A, B, and C), resulting in 21 individual samples for analysis.

### 2.2. RNA Extraction and Sequencing

RNA isolation was performed according to the basic Trizol extraction method. Then, RNA was purified, and the remaining genomic DNA was removed with Recombinant DNase I reagent (Takara, Tokyo, Japan). cDNA was synthesized and kept together with DNA at −80 °C. Polymerase chain reaction (PCR) amplification and sequencing were conducted using a novaseq PE150 platform, provided by MAGIGENE Company (Shanghai, China). Primers 515F (5′-GTG CCA GCM GCC GCG GTA A-3′) and 806R (5′-GGA CTA CHV GGG TWT CTA AT-3′) were used to amplify bacterial 16S rRNA gene sequences. Because archaeal 16S rRNA gene abundance is lower than that of bacteria in oil reservoirs, archaea have high requirements for specific primers. Thus, it was decided that nested PCR should be selected in order to enhance the sensitivity of detection for low-abundance archaea. The archaeal 16S rRNA genes were amplified using two pairs of primers Arch340F (5′-CCC TAY GGG GYG CAS CAG-3′) and Arch 1000R (5′-GGC CAT GCA CYW CYT CTC-3′), Uni519F (5′-CAG YMG CCR CGG KAA HAC C-3′), and Arch806R (5′-GGA CTA CNS GGG TMT CTA AT-3′). PCRs were performed in 50 μL mixtures and included 24 μL Premix Taq TM (Ex TaqTM Version 2.0 plus dye)/Mix [18,19].

### 2.3. Chemical Analysis

Three production wells (A, B, and C) and 21 samples were analyzed. Production water (5 mL) was centrifuged at 12,000× *g* in a high-speed centrifuge for 10 min and then filtered with membranes of 0.22 μm pore size for ion chromatography analysis. The physicochemical parameters analyzed included pH, inorganic anion and cation concentrations, and volatile fatty acid (VFA) contents [20].

### 2.4. Data Processing and Bioinformatic Analyses

Data processing was carried out on the cloud platform (http://cloud.magigene.com, accessed on 3 June 2024) according to the following steps: (1) Raw reads were filtered using fastp (v0.14.1) to remove adapters and low-quality bases. (2) Paired-end reads were merged using usearch -fastq_mergepairs (v10) with a minimum overlap of 16 bp. (3) OTUs were clustered at 97% similarity using UPARSE. (4) Taxonomy was annotated against the SILVA(16S), RDP(16S), Greengenes(16S), and SILVA (18S) databases. Sequencing was performed on the Novaseq PE150 platform (Illumina) with a read length of 150 bp for each end, and the clean reads range from 52,964 to 92,075 for bacteria and from 39,779 to 92,245 for archaea, respectively. Low-quality reads (Q20 < 90%) were filtered out before analysis. Based on OTU screening, alpha diversity indices (Chao1, Shannon) were calculated for bacterial and archaeal communities. A one-way ANOVA test was used to compare the alpha diversity indices of microbial communities at different sampling times, and post hoc pairwise comparisons used two-tailed Student *t*-tests with Bonferroni correction, carried out using GraphPad Prism software (Version 8.3.0) [21]. We used NMDS and RDA methods, which are widely used in microbial ecology to explore community–environment relationships. NMDS analysis was performed via the online analytics platform Chiplot (https://www.chiplot.online, accessed on 22 August 2024) based on Bray–Curtis dissimilarity of OTU relative abundance to visualize community differences. Redundancy analysis (RDA) was conducted in Canoco 5.0 [22], where OTU relative abundance served as the response variable and physicochemical factors (including pH, Na^+^, and CO_3_^2−^) served as explanatory variables, and significance was tested via 999 permutations. Bacterial and archaeal differences communities at the genus level were revealed using the heatmap method. Significant differences in 16S rRNA metabolic function were revealed by the extended error bar method in STAMP (STAMP Version 2.1.3) using a two-sided Fisher’s exact test [23]. We visualized community composition, diversity, and metabolic composition using Origin software (Version 2021b).

## 3. Results

### 3.1. Characteristics of Microbial Community Composition and Response to Bio-Chemical Flooding

**Figure 1:**
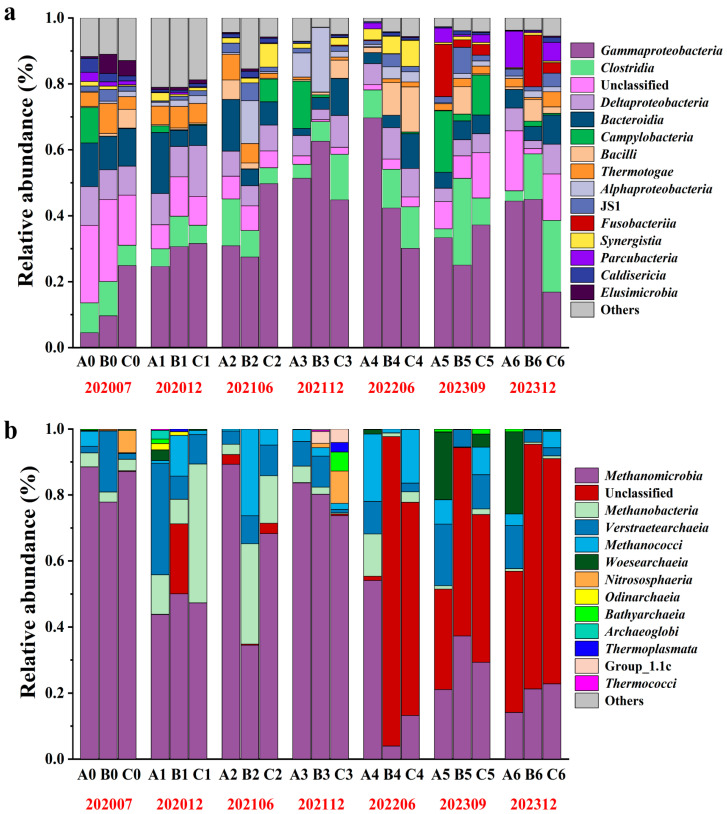
Composition of the top 15 bacterial and archaeal at the class level. (**a**) and (**b**) represent the bacterial and archaeal communities, respectively. A, B, and C at the bottom of the figure represent different sampling wells. The range from 0 to 6 represents the different sampling phases, from July 2020 to December 2023. The red data below, 202007–202312, represents the sampling periods from July 2020 to December 2023 respectively. Unclassified refers to microbial groups for which specific names cannot be determined at the current taxonomic level, and among such unclassified groups, more than 50% of the Unclassified fraction of archaea can be assigned to the phylum Euryarchaeota. The others refer to taxa with <1% relative abundance.

This study utilized cDNA-based 16S rRNA sequencing data to analyze the compositional variations in active bacterial and archaeal communities at varying injection stages (S_0_ to S_6_) in three production wells (A, B, C) during bio-chemical displacement. The investigation encompassed both the class and genus taxonomic levels. Compositions, at the class level and across seven sampling phases (S_0_ to S_6_), are shown in Figure 1. The relative abundance values of the samples from three wells at the same phase, when averaged, revealed that the dominant bacteria in the different sampling phases belonged to *Gammaproteobacteria*, *Clostridia*, *Deltaproteobacteria*, *Bacteroidia* and Unclassified bacteria, which were most frequently detected during the bio-chemical flooding injection process (Figure 1a). Furthermore, the initial two categories manifest a pronounced enrichment tendency during the displacement process. The percentage increased from 13.04% to 35.47% and from 8.52% to 12.85%, respectively. Conversely, the proportions of *Deltaproteobacteria*, *Bacteroidia*, and Unclassified bacteria exhibited a decline from 9.88%, 11.58%, and 21.19% to 6.12%, 6.24%, and 11.29%, respectively. It is noteworthy that during the late displacement phase (S_6_), the relative abundance of *Parcubacteria* (11.05%, 5.67%) in wells A and C, as well as that of *Fusobacteriia* (15.66%, 3.19%) in Wells B and C, was significantly higher than in other phases. This finding suggests that there may be a selective enrichment of specific functional groups within the local environment.

Archaea communities have been observed to demonstrate the characteristic of dominant taxa gradually replacing one another (Figure 1b). In the initial four sampling times (S_0_, S_1_, S_2_ and S_3_), *Methanomicrobia* (84.46%, 47.04%, 64.00% and 79.17%), *Methanobacteria* (3.58%, 20.51%, 15.98%, 2.50%), *Verstraetearchaeia* (7.40%, 16.54%, 7.28%, 5.99%), and *Methanococci* (1.67%, 4.80%, 10.58%, 2.68%) constitute the dominant microbial communities. In sample S_4_, a higher predominance of the relative abundance in percentages were observed for *Methanomicrobia*, *Methanobacteria*, *Verstraetearchaeia*, and *Methanococci* for Well A. It can be hypothesized that the observed phenomenon is related to the complexity of the oil reservoir. During the same time period, differences in fluid injection could lead to a heterogeneous distribution of substrates, which may select for specific archaeal metabolisms. It is hypothesized that Well A may have received a more favorable balance of substrates to support *Methanobacteria* and *Verstraetearchaeia*, whereas Wells B and C had conditions that were more conducive to the growth of *Methanomicrobia*. By the final two stages of succession (S_5_ and S_6_), the archaeal community in the reservoir had changed, with *Methanomicrobia* and Unclassified archaea now dominating. In comparison with the S_0_, an increase in the average relative abundance of Unclassified archaea was observed from 0.78% to 61.75% by the S6. Conversely, *Methanomicrobia* and *Methanobacteria* exhibited a gradual decrease from 84.46% and 3.58% to 19.35% and 0.71%, respectively. To further illustrate the changes in the dominant microbial communities over time following the introduction of the bio-chemical composite system, a line graph is presented in the Supplementary Materials (Figures S1 and S2).

**Figure 2:**
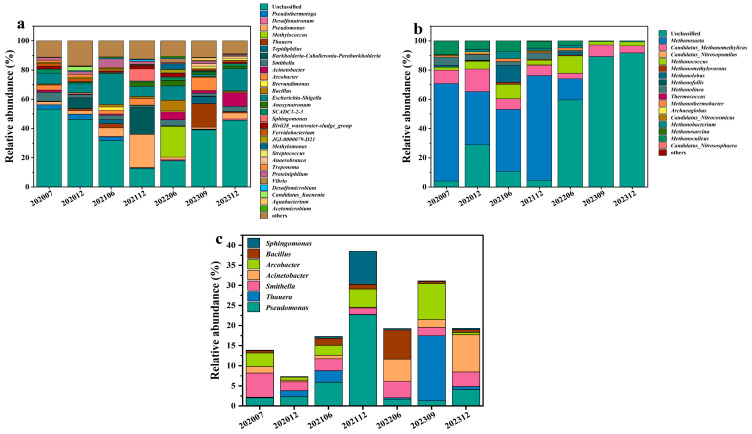
Composition of the top 30 bacterial and archaeal at the genus levels, as well as the percentage of producer biosurfactants and hydrocarbon-degrading bacteria. (**a**) and (**b**) represent bacterial and archaeal community compositions, respectively. (**c**) represents the cumulative percentage of bacteria with surfactant-producing and hydrocarbon-degrading functions within the top 30 relative abundance percentages.

The analysis of the top 30 taxonomic groups (Figure 2) further demonstrates the directed selection effect of bio-chemical displacement on functional microbial communities. Prior to injection (S_0_), the predominant genera were Unclassified archaea (53.18%), *Escherichia-Shigella* (7.50%), and *Smithella* (6.08%). Following injection, the relative abundance of *Pseudomonas* (22.69%) in S_3_ and *Methylococcus* (20.92%) in S_4_ exceeded that of Unclassified (12.78% and 17.85%) as the dominant genera. The relative abundance of *Desulfonatronum*, *Pseudomonas*, *Thauera, Acinetobacter*, and *Bacillus* increased gradually from 0%, 2.02%, 0.13%, 1.56%, and 0.58% prior to the injection of the combined flooding system to 0.73%, 4.08%, 0.79%, 9.22%, and 0.68%, respectively. Unclassified archaea, *Pseudothermotoga*, and *Smithella* decreased from 53.18%, 3.10%, and 6.08% to 45.60%, 0.17%, and 3.61%, respectively. Further analysis at the bacterial genus level revealed that seven genera previously reported to be capable of hydrocarbon degradation and biosurfactant production exhibited a significant increase from the second period onwards compared with prior to injection, from 13.84% to 19.33% (Figure 2a). A specialized analysis of microbial communities possessing hydrocarbon degradation and surfactant production functions revealed that following the second displacement phase (S_1_), the total abundance of seven reported functional bacterial groups increased from 13.84% to 19.33%. Among these, *Sphingomonas*, *Bacillus*, and *Pseudomonas* can all degrade hydrocarbons and produce biosurfactants (*Bacillus* and *Pseudomonas* retain biological activity at temperatures between 80 and 90 °C). In contrast, *Arcobacter*, *Acinetobacter*, *Smithella*, and *Thauera* only degrade hydrocarbons. Of these, *Pseudomonas*, *Thauera*, and *Acinetobacter* were identified as belonging to the *Gammaproteobacteria* class (Figure 2c).

In contrast to the evolution of bacterial communities, archaeal communities demonstrated a more straightforward evolution of their genus level composition. As shown in Figure 2b, *Methanosaeta* (66.70%), *Methanoculleus* (9.41%), and *Candidatus_Methanomethylicus* (9.15%) were the predominant archaeal genera prior to system injection. After system injection, all three genera of the archaea exhibited a downward fluctuation. The relative abundance of *Methanosaeta*, *Methanoculleus*, and *Candidatus_Methanomethylicus* decreased gradually from 66.7%, 9.41, and 9.15% to 0.008%, 0.03%, and 4.77%, respectively. Meanwhile, the Unclassified archaea category increased gradually from 4.04% (S_0_) to 91.71% (S_6_).

### 3.2. Microbial Diversity and Dynamic Changes

**Figure 3:**
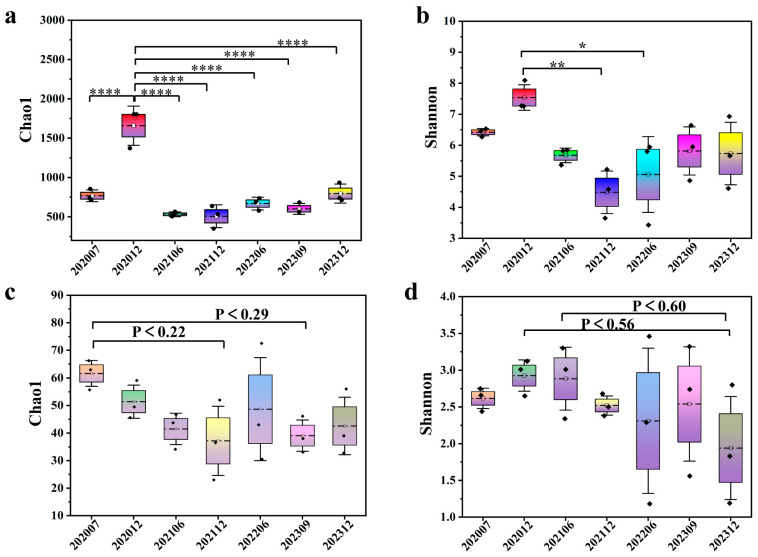
Alpha diversity including Chao1 and Shannon indices. (**a**,**b**) Bacterial communities at the OTU level. (**c**,**d**) Archaeal communities at the OTU level. A one-way ANOVA test was employed to reveal the statistical significance of the bio-chemical flooding block, and post hoc pairwise comparisons used two-tailed Student’s *t*-tests with Bonferroni correction. * represents *p* < 0.05, ** represents *p* < 0.01, and **** represents *p* < 0.0001. The horizontal axis shows the sampling periods, ranging from July 2020 to December 2023. The values on the vertical axis represent the standard deviation of measurements from three wells (A, B, and C), with error bars also indicating the standard deviation (*n* = 3).

The analysis of alpha diversities, as measured by the Chao1 index (community richness) and the Shannon index (community diversity), revealed that bio-chemical displacement exerted markedly different effects on bacterial and archaeal diversity (see Figure 3). The number of reads, operational taxonomic units (OTUs), and microbial alpha diversities were calculated within different phases under flooding processes (Table S1). There were obvious changes in the alpha diversity indices of bacteria and archaea with the injection process, as measured via a one-way ANOVA test. The Chao1 indices for S_0_ and S_6_ were comparable, while S_2_ clustered with S_3_ and S_4_ with S_5_ (similar abundance), with only S_1_ showing significant differences from other stages (*p* < 0.0001, Figure 3a). The Shannon index demonstrated a peak at S_1_ before declining to its lowest point at S_3_ (significantly lower than S_1_, *p* < 0.001), subsequently stabilizing (Figure 3b). This finding suggests that the impact of bio-chemical displacement on bacterial diversity was concentrated during the initial injection phase, with the community subsequently entering a new steady state. A significant decrease in the Chao1 and Shannon indices of the archaea was observed in comparison with those prior to injection (Figure 3c,d). The highest Chao1 index average value was 61.63 (S_0_), followed by 51.40 (S_1_), and the Chao1 index decreased to 42.57 in the final phase. The highest Shannon index average value was 2.93 for S_1_, followed by S_2_ (2.88) and S_0_ (2.62), and the Shannon index decreased to 1.94 in the final phase. This is indicative of the relative sensitivity of archaeal communities to changes in displacement environments.

**Figure 4:**
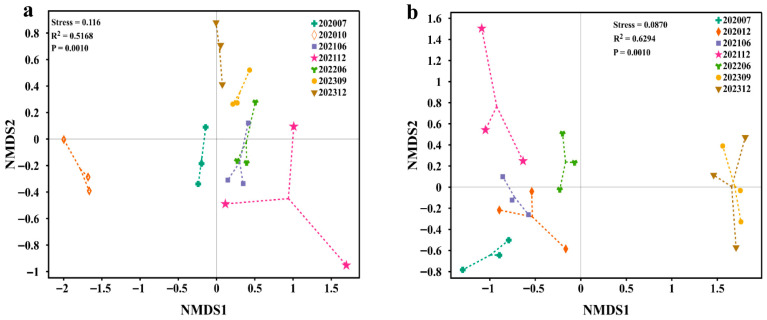
Non-metric multidimensional scaling analysis (NMDS). (**a**) Bacterial and archaeal (**b**) community structures in the different production liquids at the OTU level (assessed by Bray–Curtis dissimilarity).

In order to further explore the similarity between the microbial composition at different injection phases, non-metric multidimensional scaling (NMDS) ordination, based on Bray–Curtis dissimilarity, was applied (Figure 4). For the bacterial compositions in the samples, the three samples from S_0_ are distant from the S_6_ samples. This result indicates that the microbial diversity in the reservoir has been altered following long-term injection of bio-chemical compounds. And the S_2_, S_3_ and S_4_, S_5_, and S_6_ values were closer to one another. This finding suggests that shifts in microbial diversity occur over a protracted period. It is worth noting that, after injection, S_1_ exhibited the greatest divergence from samples of all other phases (Figure 4a). In terms of archaeal community in different phases, after the injection of the flooding systems, samples S_0_, S_1_, S_2_, S_3_, S_4_, S_5_, and S_6_ were clustered in three different quadrants (Figure 4b). Furthermore, the communities in samples S_5_ and S_6_ were distinctly separated from those of the other samples.

According to the changes in the compositions of bacteria and archaea and the results of NMDS analysis, it is clear that the composition of microorganisms in samples S_0_, S_4_, and S_6_ and the distance between the samples showed significant differences. Therefore, phase 1 (July 2020 to December 2020), phase 4 (December 2021 to June 2022), and phase 6 (September 2023 to December 2023) were selected for a comparative analysis of the differences in archaea and bacteria.

**Figure 5:**
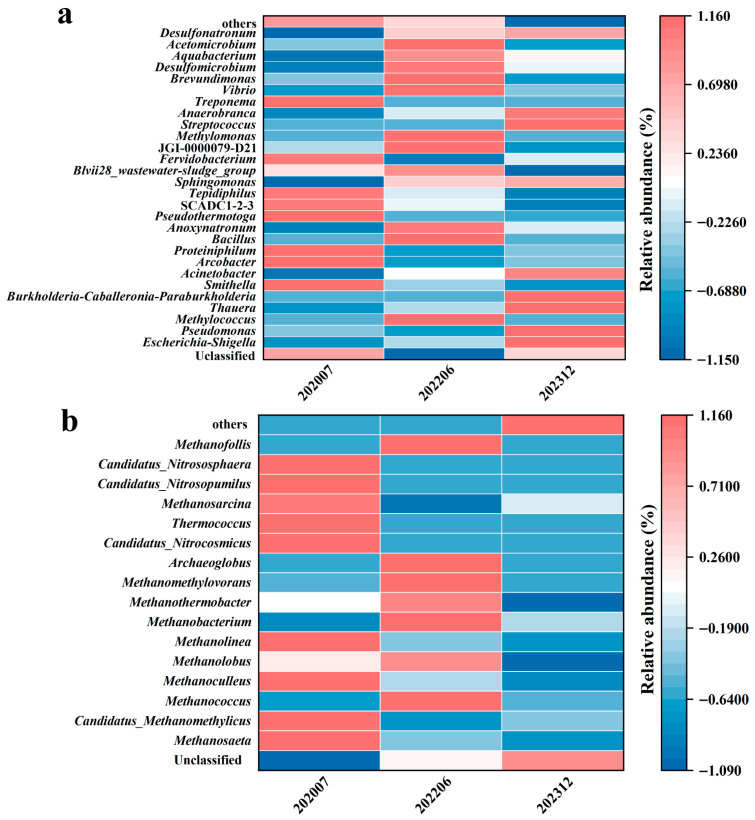
Bacterial and archaeal community differences at the genus level. (**a**) represents the results of the analysis of differences in bacteria, and (**b**) represents the archaea. The color red, ranging from pale pink to deep crimson, is indicative of an increase in the relative abundance of a genus at a specific point in time. The blue, ranging from pale blue to deep indigo, is indicative of a decrease in relative abundance. The color scale on the right quantifies this change (with values approximately ranging from −1.15 to 1.16 and −1.090 to 1.16, corresponding to the degree of reduction or increase).

The heatmap (Figure 5) illustrates the differential variation in the relative abundance of bacterial and archaeal communities at three time phases (July 2020, June 2022, and December 2023) during the injection of the bio-chemical composite flooding system. The results show that *Pseudomonas* exhibits a pronounced upward trend in abundance, particularly evident in December 2023 (deep red), suggesting significant proliferation of this genus as bio-chemical displacement progresses. Meanwhile, the genera *Thauera* and *Acinetobacter* also display sustained enrichment at later time points, as indicated by deeper red hues, and since these genera are generally linked to hydrocarbon degradation or environmental adaptation, their increase may reflect adaptation to the displaced environment. In contrast, *Tepidiphilus* and *Acetomicrobium* show a deepening of the blue hue over time, indicating a decline in their abundance as displacement continues. Notably, in the early phase (July 2020), the community contained a greater number of Unclassified taxa, but as time went on, taxa such as *Pseudomonas* (with potential hydrocarbon degradation or biosurfactant-producing functions) and *Thauera* (involved in organic matter cycling) became dominant groups (Figure 5a). There are relatively significant differences in the community composition of archaeal groups among the three phases. Genera such as *Methanosaeta*, *Methanoculleus*, *Methanolobus*, and *Methanolinea* all had relatively high proportions in the initial stage (phase 0). However, under system injection, the number of these microbial groups gradually decreased, while the proportion of Unclassified gradually increased (Figure 5b). Overall, the differences in bacterial community across different phases indicate that the injection of the bio-chemical composite system gradually altered the microenvironment of the oil reservoir and selected for specific microorganisms adapted to the reservoir conditions.

### 3.3. Influence of Physicochemical Factors on Microbial Community

**Figure 6:**
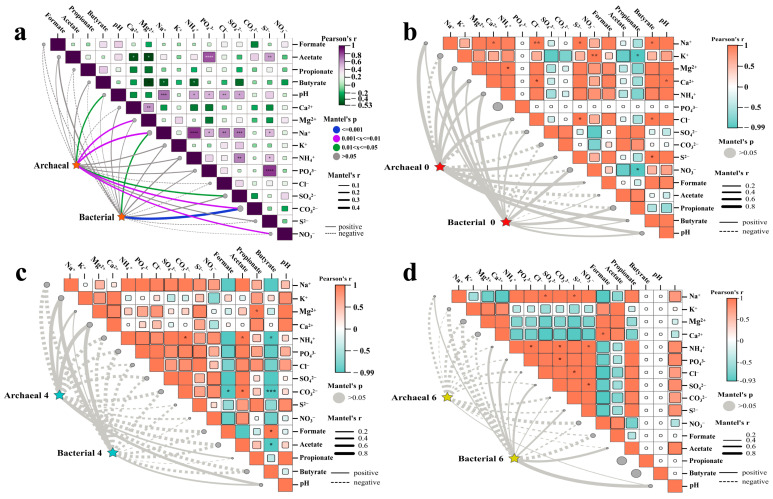
Mantel tests showing the Pearson’s correlations between bacterial and archaeal composition with physicochemical factors. (**a**,**b**,**c**) and (**d**) represent the correlation of environmental factors with bacteria and archaea during injection, and with periods 0, 4 and 6, respectively. Square size represents the significant correlations of *p*. The shade of the color represents the value of the correlation coefficients. Line width corresponds to Mantel’s r statistic for the corresponding distance correlations. The dotted line represents a negative correlation, while the solid line represents a positive correlation. * represents *p* < 0.05, ** represents *p* < 0.01, *** represents *p* < 0.001, and **** represents *p* < 0.0001.

All the physicochemical characteristics of the production waters from the block subjecting to the bio-chemical flooding in Daqing Oilfield are shown in Table S2. Pearson’s correlation and Mantel’s statistic methods were employed to evaluate the significance and correlation between the physicochemical factors and the bacteria and archaea. The influence of physicochemical factors on the dynamic changes of total bacterial and archaeal communities across the entire flooding process (integrating S_0_ to S_6_ samples) is illustrated in Figure 6a. The 10 factors demonstrated a positive correlation with changes in bacterial community, with the correlation between ions of Na^+^ (*p* < 0.05) and CO_3_^2−^ (*p* < 0.001) and bacterial communities reaching a significant level. The impact of physicochemical factors on the archaeal community change was more significant; the 12 physicochemical factors demonstrated a positive impact, with the correlation between changes in pH, Mg^2+^, Na^+^, SO_4_^2−^, CO_3_^2−^, and NO_3_^−^ reaching a significant level (*p* < 0.01, *p* < 0.01 *p* < 0.01, *p* < 0.01, *p* < 0.05, and *p* < 0.05, respectively).

A separate correlation and significance analysis of the physicochemical factors with their bacterial and archaeal communities within each individual phase (for samples S_0_, S_4_, and S_6_, respectively) indicated that the effects of the physicochemical factors on the bacterial and archaeal communities did not reach a statistically significant level in any of the three phases. In sample S_0_, archaea demonstrated a positive correlation with nine physicochemical factors, showing correlation with ions of Na^+^ (0.93), Ca^2+^ (0.90), Cl^−^ (0.93), S^2−^ (0.95), small molecules of formate (0.99), and butyrate (0.96). Bacteria were positively correlated with 13 physicochemical factors, with the highest correlation observed with ions of NH_4_^+^ and NO_3_^−^ at 0.80 and 0.79, respectively (Figure 6b). In the S_4_ samples, the types of ions and small molecular acids that exhibited a positive correlation with changes in bacteria (4) and archaea (5) were similar. Specifically, K^+^ and Ca^2+^ demonstrated a correlation with bacteria and archaea at a level exceeding *r* > 0.90 (Figure 6c). In sample S_6_, only pH was positively correlated with archaea, and nine physicochemical factors were positively correlated with bacteria, with the largest correlation being pH (*r* > 0.99) (Figure 6d).

**Figure 7:**
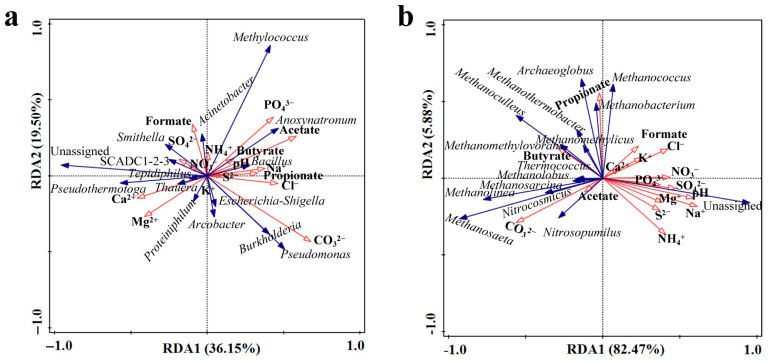
Redundancy analysis (RDA) showed the relationship between the environmental factors and the dominant bacterial (**a**) and archaeal (**b**) communities. Blue arrows represent bacterial and archaeal from different genera. Red arrows indicate environmental factors. The dashed lines primarily serve to illustrate the distribution relationships between microorganisms and environmental factors across different dimensions.

In order to explore regulatory mechanisms of physicochemical factors on functional microbial communities, redundancy analyses (RDAs) were conducted (relative abundance ≥ 0.01%, top 15 dominant genus community). Based on the results of the RDA (Figure 7a), it was shown that CO_3_^2−^ (*p* < 0.01), S^2−^ (*p* < 0.01), propionate (*p* < 0.05), PO_4_^3−^ (*p* < 0.05), and acetate (*p* < 0.05) reached a significant level for the bacterial community. Among them, CO_3_^2−^ showed a highly significant positive correlation with *Pseudomonas* and *Bacillus*, which significantly increased or decreased during system injection, as well as a significant negative correlation with *Smithella*, *Thauera*, Unclassified bacteria, and *Pseudothermotoga*. The Na^+^ (*p* < 0.01), CO_3_^2−^ (*p* < 0.01) and pH (*p* < 0.05), for the archaeal community, reached a significant level (Figure 7b). The pH and Na^+^ were found to be positively correlated with a significantly increased relative abundance of Unclassified archaea and negatively correlated with significantly decreased *Methanoculleus* among the archaea. In contrast, CO_3_^2−^ was observed to show a significant positive correlation with significantly decreased *Methanosaeta*. The aforementioned results, when considered alongside the findings of Figure 6a, indicate that the primary factors influencing alterations in bacterial and archaeal communities are Na^+^, CO_3_^2−^, and pH value.

### 3.4. Metabolic Profiling of Microbial Communities

**Figure 8:**
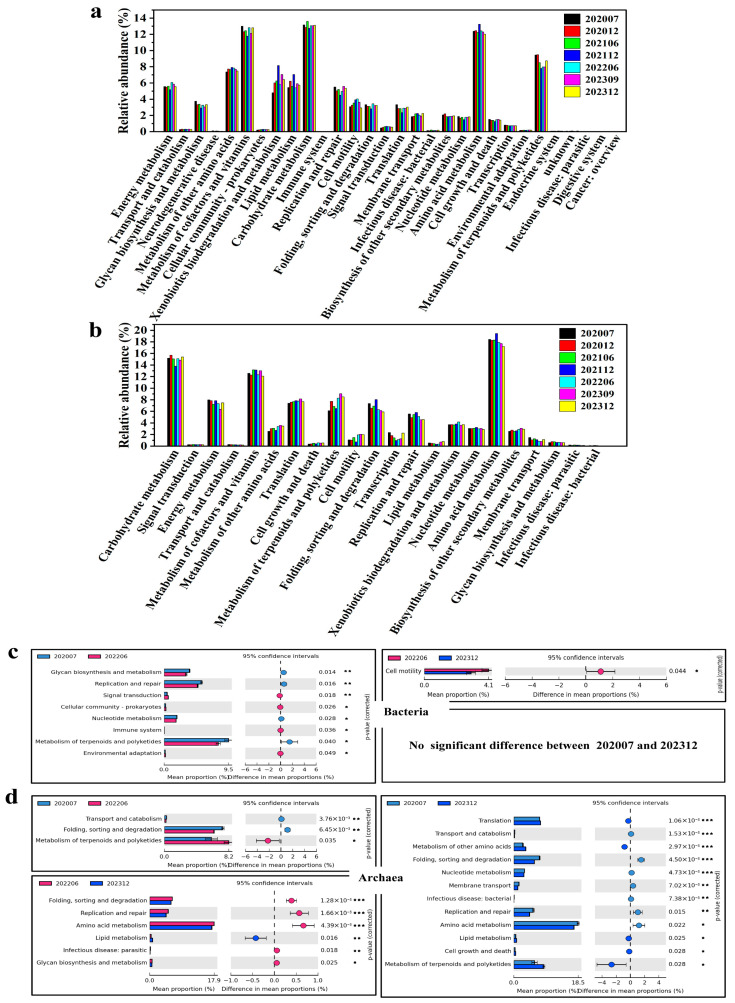
Prediction of the differential function of bacterial and archaeal communities. Relative abundances of predicted genes from bacterial (**a**) and archaeal (**b**) communities involved in the active communities from the production water at the KEGG level 2 categories. Bacterial (**c**) and archaeal (**d**) significant differences, as revealed by the extended error bar method in STAMP using Welch’s *t*-test. * represents *p* < 0.05, ** represents *p* < 0.01, and *** represents *p* < 0.001.

A functional prediction analysis was conducted on the basis of KEGG Level 2 (Figure 8). This analysis revealed adaptive changes in microbial metabolic pathways in response to bio-chemical displacement. Overall, 30 KEGG pathways were found in bacterial gene analyses, and 22 KEGG pathways were found in archaeal gene analyses. The predominant metabolic pathways of bacteria and archaea have been shown to share commonalities, with both centered on carbohydrate metabolism (bacteria 12.75–13.61%, archaea 13.81–15.69%), amino acid metabolism (bacteria 12.04–13.25%, archaea 17.20–19.43%), and metabolism of cofactors and vitamins (bacteria 11.79–13.00%, archaea12.10–13.16%) (Figure 8a,b). It is evident that metabolic pathways which were not deemed essential for survival—for example, transport and catabolism, cellular community-prokaryotes and environmental adaptation, etc.—were maintained at a relatively low level. However, it was observed that the archaeal pathways of translation (7.42–8.16%), folding, sorting and folding, and sorting and degradation (5.92–8.04%) accounted for a significantly higher proportion than in bacteria, while amino acid metabolism, exogenous biodegradation, and lipid metabolism accounted for a lower proportion than in bacteria. This finding indicates that archaea appear to demonstrate a heightened demand for the protein synthesis degradation pathway in displacement environments, in contrast to bacteria, which exhibit a pronounced emphasis on energy metabolism and the degradation of exogenous substances.

The functional differences between bacteria and archaea were compared between phase 1, phase 4, and phase 6 after each injection process, respectively. Eight metabolic functions exhibited notable differences between phase 1 and phase 4, with glycan biosynthesis and metabolism, replication and repair, and metabolism of terpenoids polyketides being more dominant (Figure 8c). In phase 4, signal transduction and nucleotide metabolism increased more significantly. Only cell motility exhibited a significant difference between phases 4 and 6 after injection. No significant difference in metabolic function was found between phase 1 and phase 6. This indicates that bacterial metabolic functions had undergone adaptive adjustments by the mid-phase (S_4_). A further analysis of the metabolic functions of the archaea in phase 1 and phase 4 indicated that three metabolic functions exhibited notable differences (Figure 8d). Among them, the metabolism of terpenoids and polyketides was significantly enhanced in phase 4, while folding, sorting, and degradation metabolism were little stronger in phase 1. Six metabolic functions were significantly different when comparing phase 4 with phase 6, of which only lipid metabolism increased to significance in phase 6. A comparison of phase 1 and phase 6 indicated significant alterations in the metabolic pathways. A total of twelve metabolic pathways were altered. Amino acid metabolism, the predominant type of metabolism in both phases, was significantly reduced in phase 6 (*p* < 0.05), with translation, metabolism of other amino acids, and lipid metabolism being significantly increased.

## 4. Discussion

Petroleum reservoirs, as unique extreme ecosystems in the deep biosphere, undergo significant shifts in microbial communities under long-term enhanced oil recovery technologies involving chemical and biological constituents [24,25]. This study set out to investigate the dynamic changes in bacterial and archaeal communities in water from three wells across six injection phases under bio-chemical flooding. The study revealed distinct patterns of community succession, environmental drivers, and metabolic adaptations.

Most previous studies on microbial composition and diversity have focused on oil reservoirs experienced water flooding, MEOR, ASP, etc. [26,27,28,29]. However, there is still a lack of knowledge about the impact of long-term flooding on the microorganisms in oil reservoirs. We used high-throughput 16S rRNA sequencing from cDNA to examine microbial dynamics under bio-chemical flooding. Before injection, *Gammaproteobacteria* and Unclassified bacteria were dominant bacterial classes. As the system continues to be injected, *Gammaproteobacteria* became the most dominant class in each injection phase (Figure 1a), consistent with the findings of previous studies on post-polymer-flooded reservoirs with nutrient stimulation [30] but different from polymer-flooded reservoirs and ASP-flooded reservoirs [9]. The relative abundance of *Gammaproteobacteria* as the active dominant taxon increased significantly. This change is not a passive fluctuation of the total community but an active response of its metabolic activity to adapt to the oil displacement environment (e.g., high pH, polyacrylamide substrate), which directly reflects the functional involvement of this taxon in polymer degradation and hydrocarbon transformation. This community shift can be directly attributed to the specific chemical components introduced during flooding. The alkaline agent (Na_2_CO_3_) elevated the reservoir pH, creating a selective pressure that favored alkali-tolerant taxa, while the injected polyacrylamide provided a novel substrate for specialized hydrocarbon-degrading bacteria.

It is noteworthy that the relative abundances of *Pseudomonas*, *Methylococcus*, *Thauera*, and *Acinetobacter* have increased, and all these genera belong to *Gammaproteobacteria* (Figure 2a,c). This enrichment can be attributed to the compatibility of *Gammaproteobacteria* with the bio-chemical flooding environment. Firstly, it has been reported that *Pseudomonas* and *Acinetobacter* exhibit strong pH tolerance [9]. Secondly, *Acinetobacter* sp. isolated from oil reservoir environments has been shown to be directly involved in the biodegradation of hydrolyzed polyacrylamide to reduce HPAM viscosity [31]. Given that the injected bio-chemical system in this study contained Na_2_CO_3_, which elevates reservoir pH, and polyacrylamide, these environmental conditions specifically stimulated the active response of its metabolic activity of bacteria within *Gammaproteobacteria*. The surge in these genera provides a clear mechanistic link: the injected polymers selected for taxa like *Acinetobacter* with direct polymer-degradation capabilities, while the elevated pH from Na_2_CO_3_ selected for pH-tolerant genera like *Pseudomonas*. Furthermore, the surfactants likely increased hydrocarbon bioavailability, promoting the growth of surfactant-producing and hydrocarbon-degrading genera like *Thauera* and *Pseudomonas*. Among them, *Pseudomonas*, *Thauera*, *Acinetobacter*, and *Arcobacter* are reported as bacterial groups important for oil recovery, and they are capable of degrading compounds or producing active surfactant to emulsify crude oil and reduce oil–water interfacial tensions [32,33,34,35,36]. *Thauera* was reported to be able to reduce nitrate and degrade aromatic compounds [37,38]. *Methylococcus* is capable of utilizing methane as the sole carbon source and energy to produce carbon dioxide and water, thereby contributing to the global carbon cycle. *Sphingomonas* is able to degrade hydrocarbons and produce microbial exopolysaccharide [39].

In contrast, archaea exhibited greater sensitivity to injections, with a shift from *Methanomicrobia* dominance to high proportions of Unclassified classes in later phases (Figure 1b). This transition coincided with declining abundances of acetoclastic methanogens *Methanosaeta* and CO_2_-reducing methanogens *Methanolinea* and *Methanoculleus* (Figure 2b) [40,41,42,43]. The increase in the proportion of Unclassified archaea can be attributed to two main reasons. Firstly, while 16S rRNA gene sequence analysis is an important method for archaeal classification, it has limitations in terms of classification at the genus level. Secondly, the increase may be due to the unique environment of long-term bio-chemical flooding, which selects for novel archaeal taxa not yet classified in existing databases. Concurrently, a decline in *Methanosaeta* was evident, which might be attributable to the enrichment of active acetate-utilizing bacteria (e.g., *Acinetobacter*) intensifying competition for acetate. Our metabolite analysis (Supplementary Table S2) showed that reservoir acetate concentration decreased from 1.1 to 12.4 mg/L (S_0_) to 0.34–0.78 mg/L (S_4_). It has been reported that competition between different prokaryotes for substrates such as formate and carbon dioxide indicates that microorganisms compete for shared substrates within ecosystems [44]. Since *Methanosaeta* has a lower affinity for acetate than *Acinetobacter*, it is outcompeted for this key substrate, further suppressing its metabolic activity. This demonstrates a key mechanism of chemical impact. Injected chemicals altered the substrate microenvironment, triggering competitive interactions that affected archaeal methanogens.

Alpha diversity analyses indicated that the bacterial abundance in the last phase did not change significantly compared to those of prior to injection, with diversities decreasing under the flooding operation (Figure 3a,b). It seems that injecting a combination of both biological and chemical constituents into the flooding systems only enriched some microbial populations, resulting in selectivity of the dominating species in whole communities, consistent with Gao et al., who attributed such patterns to the dominance of stimulated populations masking low-abundance taxa [30]. Archaeal diversity, however, declined significantly, potentially due to their narrower ecological niches and sensitivity to chemical perturbations (Figure 3c,d). The NMDS analysis demonstrated that both the bacterial and archaeal populations experienced successional changes in response to bio-chemical flooding system injection, particularly the distinct clustering of phase 5 and 6 samples of archaea, confirming that long-term bio-chemical flooding drives directional community restructuring, leading to a new ecological balance (Figure 4a,b). Differential analysis reveals that bacterial and archaeal types undergo dynamic changes with the injection of bio-chemical flooding systems (Figure 5).

Physicochemical factors also influence the diversity and composition of microbial communities inhabiting the subsurface oil reservoirs, such as the chemical composition of the formation brine, pH, permeability of oil formations, and other stochastic processes [27,45,46,47]. Our results provide a strong and explicit correlation between these physicochemical changes induced by flooding and the observed microbial shifts. Figure 6a shows CO_3_^2−^ had a highly significant effect on bacteria (*p* < 0.001). RDA further revealed that dominant genera like *Pseudomonas*, *Escherichia*-*Shigella*, *Arcobacter*, and *Burkholderia* were significantly positively correlated with CO_3_^2−^, while *Smithella*, *Acinetobacter*, and Unclassified bacteria were significantly negatively correlated (Figure 7a). Among them, *Pseudomonas*, *Arcobacter*, *Smithella*, and *Acinetobacter* possessed the capability to degrade petroleum hydrocarbons, produce surfactants, and degrade polyacrylamide [35,48]. *Acinetobacter*’s negative correlation with CO_3_^2−^ contrasts with its known adaptability to saline and anaerobic niches. It has been reported in preceding studies that *Acinetobacter* serves as a key hydrocarbon degrader in anaerobic saline oil reservoirs, suggesting context-dependent responses to carbonate ions in our specific bio-chemical flooding system [49,50]. This correlation analysis directly links the chemical agent (Na_2_CO_3_, CO_3_^2−^) to the enrichment of specific bacterial genera, illustrating a mechanism by which injected chemicals shape the community.

RDA of archaea indicated that CO_3_^2−^ exhibited a positive correlation with *Methanosaeta*, a highly variable group of archaea (Figure 7b). pH and Na^+^ were the crucial physicochemical factors that can markedly influence the composition of archaeal communities across a diverse range of habitats. The only environmental factor that demonstrated a positive correlation with archaea in phase 6 was pH (Figure 6d). Similarly, the single factor with the greatest correlation with the bacteria was also pH (*r* > 0.99). In light of the impact of CO_3_^2−^ alterations on the bacterial within oil reservoirs, particularly those that play a pivotal role in oil recovery, it is evident that the acidity and alkalinity of the reservoir environment may serve as a crucial determinant in the distribution of microorganisms. This finding is consistent with previous results, which demonstrated a strong correlation between bacterial community distribution and pH [51,52]. This relationship also offers valuable insights into the structural and functional characteristics of microbial communities under diverse flooding ways. The paramount importance of pH, a direct consequence of the Na_2_CO_3_ injection, underscores a primary mechanism through which bio-chemical flooding exerts its selective pressure on both bacterial and archaeal communities.

The PICRUSt function prediction analysis based on 16S rRNA high-throughput sequencing has been widely used in the research of microorganisms [53,54]. In this study, we predicted the metabolic functions of bacteria and archaea by PICRUSt. The analysis results indicate that bacteria and archaea exhibit common characteristics with regard to metabolic pathways, including carbohydrate metabolism, amino acid metabolism, and metabolism of cofactors and vitamins. These pathways are of significant importance for microorganisms and are indispensable for their fundamental survival and proliferation (Figure 8a,b). The analysis of the differences in metabolic function at phases 1, 4, and 6 indicated that the discrepancies in bacterial metabolic processes diminished progressively during the process of biological and chemical constituent flooding injection (Figure 8c). Archaeal metabolism showed greater variability, particularly in terpenoid and polyketide metabolism—key for membrane stability and defense in extreme conditions—indicating ongoing adaptation to prolonged flooding (Figure 8d). The lack of long-term metabolic divergence in bacteria, compared to archaea, suggests faster acclimation or broader metabolic flexibility. It is currently unclear whether the changes in metabolic function types of archaea would eventually return to the pre-injection state, as in the case of the bacteria, and further long-term observation is needed [55,56]. The results of the functional prediction and analysis of microbial community structure indicate that the injected chemicals selectively enriched specific bacterial communities, driving bacterial metabolic potential towards functionally advantageous adaptations within the novel reservoir environment. Concurrently, the archaeal community exhibited a more stress-responsive behavior characterized by increased functional diversity.

## 5. Conclusions

This study analyzed the dynamic changes in oilfield microbial communities under a long-term bio-chemical flooding operation. Key findings include the selective enrichment of bacterial classes associated with hydrocarbon degradation and biosurfactant production (e.g., *Gammaproteobacteria* and *Clostridia*), as well as specific bacterial genera (e.g., *Pseudomonas* and *Thauera*). Meanwhile, archaeal communities underwent profound restructuring, with a notable decline in methanogens and an increase in Unclassified taxa. These compositional shifts were primarily driven by changes in key physicochemical factors, particularly ions of Na^+^ and CO_3_^2−^ and pH values. Furthermore, our metabolic function analysis has revealed the core metabolic composition and differences between bacteria and archaea; the bacteria emphasized energy metabolism and the degradation of exogenous compounds, whereas archaea prioritized protein synthesis and turnover. The broader significance of this work lies in elucidating the microbial mechanisms that underpin enhanced oil recovery (EOR), providing a scientific basis for designing more efficient bio-chemical flooding strategies by leveraging functional microbial consortia. Future research should incorporate metatranscriptomic or metaproteomic analyses to validate the activity of potential functional genes and verify the relationship between specific taxonomic groups and injected bio-chemical composite systems and environmental factors through laboratory experiments under controlled conditions.

## Data Availability

The original contributions presented in the study are included in the article/Supplementary Materials, and the 16S rRNA gene amplicon sequencing was submitted to the NCBI SRA database under BioProject PRJNA1208403.

## Supplementary Materials

The following supporting information can be downloaded at: https://www.mdpi.com/article/10.3390/microorganisms13102246/s1, Table S1: A, B, and C oil well reads, as well as OTU and alpha diversity indexes of the bacterial and archaeal communities during long-term flooding by a mixture of biological and chemical constituents; Table S2: Physicochemical parameters of the samples collected from different stages of biological and chemical constituents injection process; Figure S1. Composition of the top 15 bacterial and archaeal at the class levels. (a), (b), and (c) represent the bacterial community, and (d), (e), and (f) represent the archaeal community, respectively. A, B, C at the bottom of the figure represent different sampling wells. The range from 202007 to 202312 represents the different sampling phases. Unclassified refers to microbial groups for which specific names cannot be determined at the current taxonomic level, and among such groups, more than 50% of the Unclassified fraction of archaea can be assigned to the phylum Euryarchaeota. The others refer to taxa with <1% relative abundance; Figure S2. Composition of the top 30 bacterial and archaeal at the genus levels, and the percentage of producer biosurfactants and hydrocarbon-degrading bacteria. (a) and (b) represent the bacterial and archaeal community compositions, respectively. (c) represents the cumulative percentage of bacteria with surfactant-producing and hydrocarbon-degrading functions within the top 30 relative abundance percentages.
