# Supplementary material for: Dynamic Changes in Microbial Communities in Oil Reservoirs Under a Long-Term Bio-Chemical Flooding Operation
Source: Microorganisms. 2025 Sep 25;13(10):2246. doi: 10.3390/microorganisms13102246 (PMC12565960; doi:10.3390/microorganisms13102246)
Supplement: Supplementary file 1 [file microorganisms-13-02246-s001.zip › microorganisms-3838175-supplementary.pdf]

Dynamic changes in microbial communities in oil reservoirs under a long-term bio-chemical flooding operation

Gui-Na Qi [1,2], Guo-Jun Li [1,2], Yi-Fan Liu [1,3], Lei Zhou [1,3], Ya-Qing Ge [2], Jin-Feng Liu [2], Shi-Zhong Yang [1,3], Ji-Dong Gu [4] and Bo-Zhong Mu [1,3,*]

[1]State Key Laboratory of Bioreactor Engineering, School of Chemistry and Molecular Engineering, East China University of Science and Technology, Shanghai 200237, China; qiguina@mail.ecust.edu.cn (G.-N.Q.); liguojun@mail.ecust.edu.cn (G.-J.L.); liuyifan@ecust.edu.cn (Y.-F.L.); leizhou@ecust.edu.cn (L.Z.); meor@ecust.edu.cn (S.-Z.Y.)
[2]Daqing Huali Biotechnology Co., Ltd., Daqing 163000, China; shapigazi123@126.com (Y.-Q.G.); ljf6713@sina.com (J.-F.L.)
[3]MOE Engineering Research Center of Microbial Enhanced Oil Recovery, East China University of Science and Technology, Shanghai 200237, China
[4]Environmental Science and Engineering Program, Guangdong Technion—Israel Institute of Technology, 241 Daxue Road, Shantou 515063, China; jdgu.aeb@gmail.com
*Correspondence: bzmu@ecust.edu.cn; Tel: +86-21-64252063

Table S1. A, B and C oil wells reads, OTU and alpha diversity indexes of the bacterial and archaeal communities during long-term flooding by a mixture of biological and chemical constituents

Well no.	Sampling stages	Clean reads (Bacteria/Archaea)	Observed OTUs (Bacteria/Archaea)	Chao1 (Bacteria/Archaea)	Shannon (Bacteria/Archaea)
A	0	92075/84963	734/66	734.7/66.2	6.27/2.75
	1	89521/92245	1801/59	1801.4/59.1	8.09/3.12
	2	87814/87679	527/34	527.6/34.1	5.84/2.34
	3	52964/54860	532/34	532.8/52.0	4.58/2.50
	4	87188/90347	577/48	577.7/72.5	3.43/3.46
	5	84504/89938	560/43	560.8/46.1	4.86/3.32
	6	84778/87964	714/48	714.9/56.0	4.61/2.80
B	0	88411/90491	713/63	713.9/63.0	6.46/2.44
	1	83978/89100	1371/42	1371.4/45.6	7.26/3.01
	2	90473/88253	503/43	504.1/43.7	5.82/3.30
	3	57750/37308	349/24	349.9/36.5	3.65/2.38
	4	90538/86380	734/30	734.4/30.5	5.94/1.18
	5	88204/84758	681/30	682.3/38.0	6.64/1.56
	6	91042/88122	933/32	933.7/32.7	5.66/1.19
C	0	78116/84561	854/55	854.3/55.7	6.53/2.66
	1	88453/85141	1803/45	1803.3/49.5	7.27/2.65
	2	87168/90174	563/44	564.3/46.7	5.36/3.01
	3	72605/39779	636/23	636.7/23.0	5.22/2.68
	4	84282/87604	692/42	692.4/43.0	5.80/2.29
	5	88729/91855	561/30	561.6/33.1	5.95/2.74
	6	86273/86809	737/39	737.6/39.0	6.93/1.83

A, B and C represents different oil wells；0 to 6 represent different production stages from July 2020 to December 2023.

Table S2. Physicochemical parameters of the samples collected from different stages of biological and chemical constituents injection process

			**Concentration** (mg/L)														
**Well**	**Stages**	**pH**	**Ca^{2+}**	**Mg^{2+}**	**Na^{+}**	**K^{+}**	**NH_4^{+}**	**PO_4^{3-}**	**Cl^{-}**	**SO_4^{2-}**	**CO_3^{2-}**	**S^{2-}**	**NO_3^{-}**	**Formate**	**Acetate**	**Propionate**	**Butyrate**
	0	7.73	13.7	2.54	1431	3.04	17.8	0.03	603	0.764	724	0.333	0.408	0.141	12.4	0.671	0.606
	1	7.85	84.7	6.05	1800	12.7	0.6	1.64	965	6.83	550	0.1	0.1	0.23	0.04	0.18	0.5
	2	7.6	13.4	2.64	2015	6.12	3.23	0.238	985	0.05	498	0.619	0.151	0.078	1.9	3.45	1.01
**A**	3	8.17	24.1	6.87	2183	11.6	6.03	1.05	979	0.51	3037	0.02	0.06	0.03	3.71	0.03	0.25
	4	8.35	1.14	0.2	2944	7.58	22.5	11.4	1029	3.09	730	0.5	1.48	0.36	24.4	4.14	0.67
	5	8.11	40.4	11.2	2787	81.8	18	0.55	1112	15.6	115	0.05	7.43	0.19	0.91	0.09	0.01
	6	7.61	31	8.47	2904	6.23	16.3	2.52	989	1.67	87.7	0.57	1.14	0.03	0.34	0.06	0.09
	0	7.77	16.2	3.68	1450	4.41	18.7	0.03	655	0.694	701	0.36	0.757	0.065	1.1	0.079	0.637
	1	7.84	64.9	4.8	1631	4.93	0.53	0.05	819	1.36	477	0.1	0.17	0.22	0.04	0.1	0.5
	2	8.18	24.3	4.6	2650	9.1	12.9	0.247	1174	1.04	667	0.697	0.158	0.461	0.063	0.434	0.3
**B**	3	8.35	17.7	5.17	3338	10.6	27.4	3.57	1114	2.65	4118	0.42	0.07	0.04	25.2	0.03	0.3
	4	9	1.6	0.49	3615	9.77	31.2	18.6	1117	3.37	803	6.34	1.29	0.3	32	5.32	0.09
	5	9.42	21.6	4.38	4169	10.2	44.4	0.14	1129	23.2	148	0.05	7.47	0.6	1.07	0.14	0.01
	6	8.55	21.4	6.98	4767	6.58	54.9	7.61	1036	16.3	123	2.87	0.78	0.03	0.78	0.06	0.09
	0	7.88	25.9	5.6	1564	4.41	19.9	0.03	956	0.718	715	0.576	0.758	0.575	5.99	0.125	1.03
	1	8.21	93.9	7.43	1741	4.93	2.1	0.5	916	4.07	532	0.1	0.12	0.39	0.46	0.38	0.5
	2	8.27	11.6	3.02	1876	9.1	2.77	0.208	1015	0.05	490	0.734	0.063	0.067	1.91	18.9	1.14
**C**	3	8.45	11.4	3.31	2614	10.6	16.6	1.92	1012	1.04	3519	0.02	0.08	0.04	6.19	0.03	0.28
	4	8.65	1.48	0.31	2102	9.77	0.009	6.07	998	0.52	494	0.32	0.82	0.63	6.62	4.53	2.54
	5	8.01	52.5	8.55	2483	7.64	12.1	1.74	1010	7.15	124	0.05	26.8	0.41	0.7	0.27	0.01
	6	8.04	47.1	13.9	2408	8.46	14.6	2.51	974	0.63	75.4	0.28	1.64	0.09	0.38	0.06	0.09

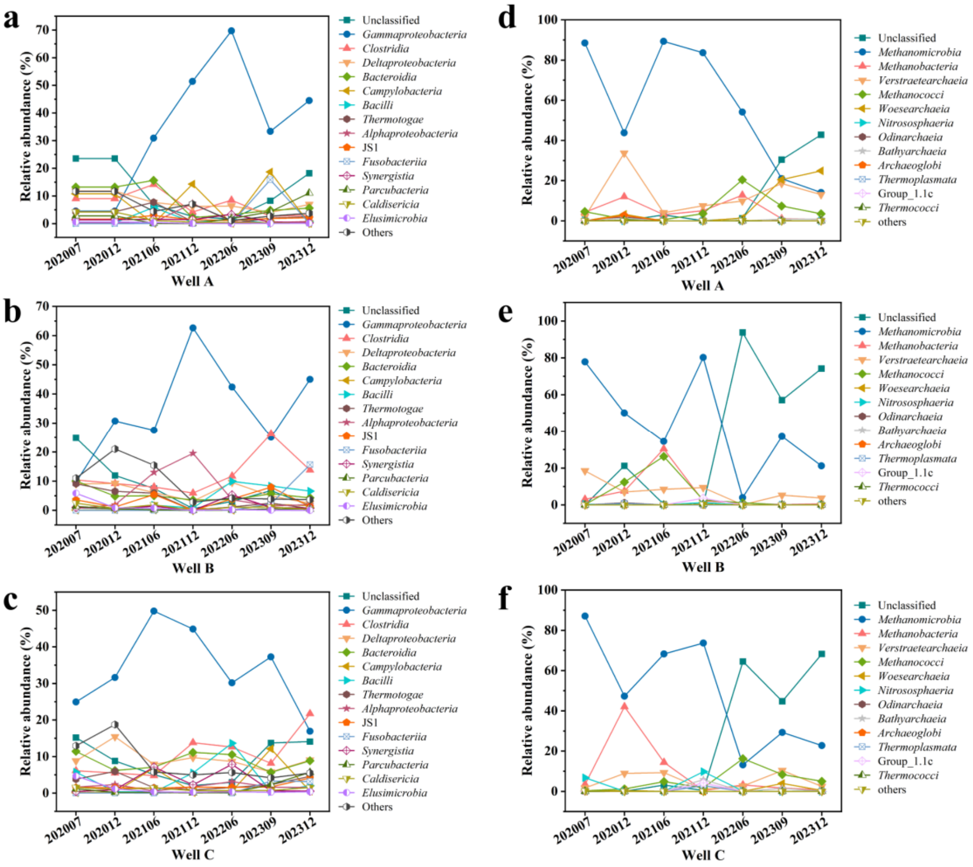

Figure S1. Composition of the top 15 bacterial and archaeal at the class levels. **(a)**, **(b)** and (c) represent the community of bacterial and **(d)**, **(e)**, **(f)** represent archaeal, respectively. (A, B, C at the bottom of the figure represent different sampling wells. The range from 202007 to 202312 represents the different phase of sampling Unclassified refers to microbial groups for which specific names cannot be determined at the current taxonomic level, and among these unclassified groups, more than 50% of the Unclassified fraction of archaea can be assigned to the phylum Euryarchaeota. The others refer to taxa with <1% relative abundance.

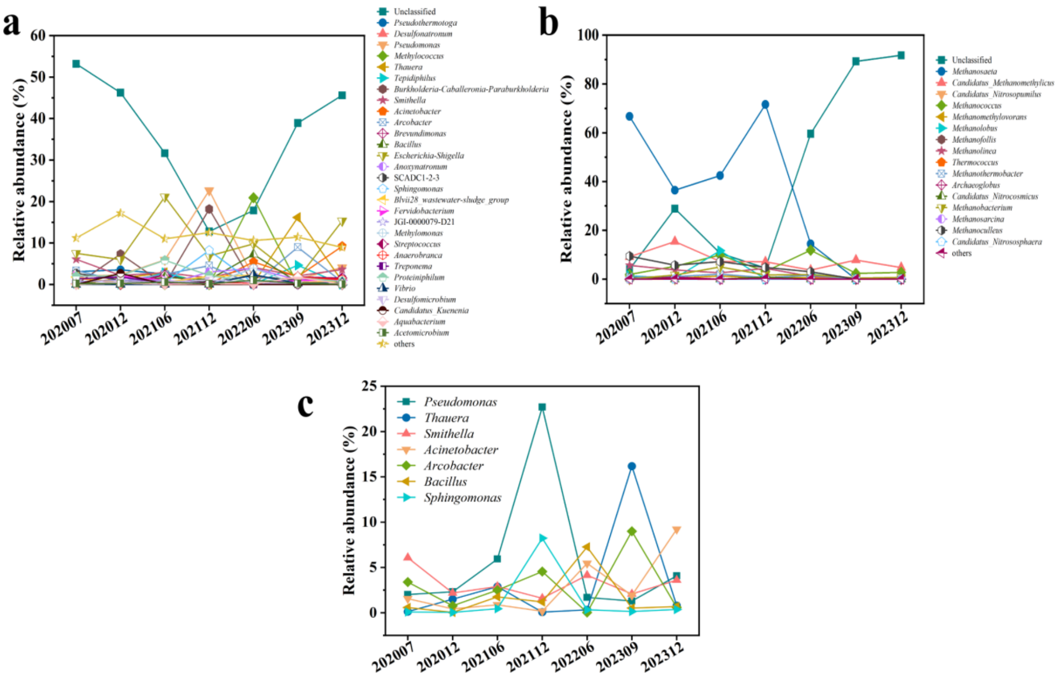

Figure S2. Composition of the top 30 bacterial and archaeal at the genus levels, and the percentage of producer biosurfactants and hydrocarbon-degrading bacteria. **(a)** and **(b)** represent bacterial and archaeal community composition, respectively. **(c)** Represents the cumulative percentage of bacteria with surfactant-producing and hydrocarbon-degrading functions within the top 30 relative abundance percentages.
